# Aurora Kinases as Druggable Targets in Pediatric Leukemia: Heterogeneity in Target Modulation Activities and Cytotoxicity by Diverse Novel Therapeutic Agents

**DOI:** 10.1371/journal.pone.0102741

**Published:** 2014-07-21

**Authors:** Aarthi Jayanthan, Yibing Ruan, Tony H. Truong, Aru Narendran

**Affiliations:** 1 Pediatric Oncology Experimental Therapeutics Investigators Consortium (POETIC) Laboratory for Pre-Clinical and Drug Discovery Studies, University of Calgary, Calgary, Alberta, Canada; 2 Division of Pediatric Oncology, Alberta Children's Hospital, Calgary, Alberta, Canada; Institut de Génétique et Développement de Rennes, France

## Abstract

Leukemia is the most common pediatric malignancy, constituting more than 30% of all childhood cancers. Although cure rates have improved greatly, approximately one in five children relapse and poor survival rates post relapse remain a challenge. Given this, more effective and innovative therapeutic strategies are needed in order to improve prognosis. Aurora kinases, a family of serine/threonine kinases essential for the regulation of several mitotic processes, have been identified as potential targets for cancer therapeutics. Elevated expression of Aurora kinases has been demonstrated in several malignancies and is associated with aberrant mitotic activity, aneuploidy and alterations in chromosomal structure and genome instability. Based on this rationale, a number of small molecule inhibitors have been formulated and advanced to human studies in the recent past. A comparative analysis of these agents in cytotoxicity and target modulation analyses against a panel of leukemia cells provides novel insights into the unique mechanisms and codependent activity pathways involved in targeting Aurora kinases, constituting a distinctive preclinical experimental framework to identify appropriate agents and combinations in future clinical studies.

## Introduction

Leukemia develops through the transformation of hematopoietic progenitor cells blocked at an early stage of differentiation, leading to uncontrolled proliferation of abnormal leukemic blasts and suppression of normal haematopoiesis, decreasing the number of mature cells in the blood [1], [2]. At present, the five year event-free survival rates for children diagnosed with acute lymphoblastic leukemia (ALL) and acute myeloid leukemia (AML) undergoing protocol-based therapy in developed countries range from 76% to 86% and from 49% to 63%, respectively [3]. Comparatively, the event free survival rates for infant leukemia, especially for infants with *MLL* rearrangements, is significantly lower compared to older children, ranging from 30% to 40% [3], [4]. Despite improved survival rates in the recent past, approximately 20% of children with ALL and 30% of children diagnosed with AML relapse [5], [6]. Of those who relapse, only 40% to 50% survive with current therapies, which include re-induction treatment and hematopoietic stem cell transplantation [6], [7]. Given the incidence of refractory and relapsed leukemia and its poor response to current available treatments, novel therapeutic approaches are being actively pursued by cooperative groups and early phase clinical trial consortia.

It has been well established that cell cycle protein kinases are overexpressed and exhibit aberrant activity in several malignancies, resulting in uncontrolled proliferation [8]–[10]. As a result, small molecule kinase inhibitors have been developed targeting these proteins. One group of cell cycle protein kinases of particular interest are the Aurora kinases. Aurora kinases are a family of serine/threonine kinases essential for regulated mitotic cell division [11]. It has been determined that these proteins are involved in regulating centromere duplication, formation of a bipolar mitotic spindle, chromosome alignment on the mitotic spindle and fidelity monitoring of the spindle checkpoint, thereby promoting genome stability [12]. At present, three aurora kinase isoforms have been identified in mammalian cells: Aurora kinase A (Aurora-A), Aurora kinase B (Aurora-B) and Aurora kinase C (Aurora-C) [13], [14]. The isoforms differ in localization, expression levels and timing of activity [15].

Elevated expression of Aurora kinases has been identified in several primary tumours types, including breast, ovarian, gastric, colon and pancreatic, among others [8]. It has been determined that corresponding gene amplification and overexpression of Aurora-A overrides the spindle checkpoint, produces aberrant chromosomes and leads to transformation [16]. Similarly, overexpression of Aurora-B leads to increased phosphorylation of histone H3 and the formation of more aggressive tumours in transgenic mouse models [17], [18]. Given that cancer cells tend to divide faster than normal cells, proteins that disrupt this process can preferentially harm cancer cells before non-tumorigenic cells in the body. The demonstrated overexpression in many forms of cancer and their involvement in mitotic control and genomic instability make Aurora kinases a promising target for therapeutics. It is important to note that Aurora kinase inhibitors do not induce mitotic arrest like antimitotic agents. Rather, these inhibitors promote aberrant mitosis, leading to arrest in a pseudo G1 state and multiple cell cycles without cytokinesis, resulting in a polyploid phenotype [19]. These factors contribute to the induction of mitotic catastrophe, considered to be a cell death mechanism caused by aberrant mitosis, leading to apoptosis [20].

The majority of Aurora kinase inhibitors developed to date target the ATP binding site and are either pan-Aurora inhibitors or selective Aurora-A or Aurora-B inhibitors [21]. Most often, cells exposed to dual Aurora-A/Aurora-B inhibitors express phenotypes indicative of Aurora-B inhibition [22]. Aurora kinase inhibitors may have significant advantages over traditional inhibitors targeting mitosis, such as taxanes and vinca alkaloids, which target microtubules. There are dose limiting toxicities associated with these conventional therapies, as tubulin is essential for several cellular processes in addition to mitosis [23]. Although it has been established that several Aurora kinase inhibitors induce apoptosis, details of the mechanisms of these processes are currently unclear and are the subject of investigation in a number of laboratories. The availability of a spectrum of Aurora kinase inhibitors with targeted but distinct activities provide a unique opportunity to uncover molecular interrelationships and associated pathways of control. The objective of this study is to assess, in preclinical studies, the activity of Aurora kinase inhibitors against pediatric and infant leukemia cell lines and primary samples with respect to target availability and off-target effects. We also aim to evaluate the heterogeneity in activity of the different Aurora kinase targeting agents in the context of distinct molecular vulnerabilities seen among pediatric leukemia subgroups.

## Materials and Methods

This study was approved by: Conjoint Health Research Ethics Board, Calgary Region and the University of Calgary. Peripheral blood and bone marrow samples were obtained from infants and children diagnosed with leukemia at the Alberta Children's Hospital after ethics approval from the Conjoint Health Research Ethics Board and written informed consent from parents and assent as appropriate (Ethics ID #17184). Normal peripheral mononuclear cells were obtained from healthy individuals with signed informed consent.

### Cell lines and tissue culture

Cell lines were cultured in Opti-MEM medium (Gibco, Life Technologies Corporation, Burlington, Ontario) supplemented with 10% heat inactivated fetal bovine serum (FBS) (Gibco), 100 units/ml penicillin and 100 units/ml streptomycin (Gibco) and 0.05 mM 2-mercaptoethanol (Sigma-Aldrich, Oakville, Ontario) in T25 flasks (Nalge Nunc, Rochester, New York) or T75 flasks (Corning Incorporated, Corning, New York). All cell cultures were maintained at 37°C in a humidified incubator with 5% CO_2_. Characteristics of the cell lines used in this study are summarized in Table S1.

### Isolation Of White Blood Cells From Peripheral Blood And Bone Marrow Samples

Peripheral blood and bone marrow samples were obtained from infants and children diagnosed with leukemia at the Alberta Children's Hospital after local Institutional Review Board approval and informed consent and assent as appropriate (Ethics ID #17184). These include B-ALL (Patient #1), relapsed AML (Patient #2), AML/CML (Patient #3), infant ALL (Patient #4), and initial ALL presenting with secondary AML (Patient #5). The samples were diluted to double its original volume with sterile phosphate buffered saline (PBS; 137 mM NaCl, 2.7 mM KCl, 10 mM Na_2_HPO_4_, 1.8 mM KH_2_PO_4_; pH 7.25). The diluted sample was then layered on a Ficoll-Paque (GE Healthcare, Piscataway, New Jersey) gradient at a 1∶2 ratio of Ficoll to diluted sample. Samples were then centrifuged 1800 rpm for 20 minutes at room temperature. The buffy coat was removed with a pipette and transferred to a new centrifuge tube and diluted with PBS. After an additional centrifugation at 1200 rpm, the white blood cell pellet was washed with PBS and centrifuged one last time. Cells were re-suspended in Opti-MEM and viable cells were counted using trypan blue (Invitrogen, Life Technologies Corporation, Burlington, Ontario) exclusion on a haemocytometer. An identical protocol was followed for the isolation of white blood cells, referred to as “normal lymphocytes” in the following paragraphs, from healthy volunteers. Samples grown in culture for up to six months were cultured in Opti-MEM medium supplemented with 10% heat inactivated FBS, penicillin/streptomycin and 2-mercaptoethanol. Characteristics of primary samples used in this study for cytotoxicity assays are summarized in Table S2.

### Aurora Kinase Inhibitors

A library of fourteen inhibitors targeting Aurora kinases was provided by Selleck Chemicals (Houston, Texas) and included: PHA-739358, PHA-680632, CYC116, CCT129202, Aurora A Inhibitor I, ZM447439, JNJ-7706621, ENMD-2076, MLN8237, SNS-314, AZD1152, Hesperadin, AT9283 and VX-680. All of the inhibitors were prepared in DMSO to a concentration of 10 mM and stored in 10 µl aliquots at −20°C. For subsequent experiments, the inhibitors were diluted in Opti-MEM plus supplements to the appropriate concentrations.

### Cytotoxicity Assays

For cytotoxicity profiling of Aurora kinase inhibitors against leukemia cell lines, inhibitors or vehicle control (DMSO, Sigma-Aldrich) were diluted in 100 µl of Opti-MEM per well in 96 well plates (Grenier Bio-One, Monroe, North Carolina) in triplicate at final concentrations ranging from 1×10^−6^ to 10 µM. 1×10^4^ cells per well were seeded and plates were incubated for 4 days. Following incubation for 4 days, cell survival was quantified by Alamar blue assay (Invitrogen) or automated inverted microscopy using the Celigo Cell Cytometer (Cyntellect, San Diego, California). For Alamar blue assay, cells were incubated with 2.5% Alamar blue, which incorporates a propriety redox indicator that changes colour in response to metabolic activity, for up to 24 hours. During this period of incubation, the absorbance at 570–620 nm was measured (Opsys MR Plate Reader, Dynex Technologies, Chantilly, Virginia) at various time points, depending on the cell line or sample. Percent survival was calculated by comparing the absorbance ratio of the test well to the control well multiplied by 100%, as indicated in the following formula: 

For automated inverted microscopy, the number of cells in each well was determined using the direct cell counting application as described previously [24]. Briefly, following adjustment of image acquisition settings for bright-field imaging, the wells containing cells and appropriate dilutions of inhibitors were individually scanned. Based on these images, analysis parameters, including intensity threshold and cell diameter, were modified for optimal inclusion of viable cells in treated and control wells. These parameters were applied for subsequent replicates involving the same leukemia cell line or sample. The percent survival was then calculated as a ratio of the number of cells in treated wells to the number of cells in control wells multiplied by 100%. Given that Aurora kinase inhibitors interfere with cytokinesis, cells that are committed to death may still contain enlarging but intact cytoplasmic content before the eventual loss of cytoplasmic membrane integrity. The accuracy of Alamar blue assay may be, in theory, affected by the timing of these events. For this reason, both techniques were employed to determine Aurora kinase inhibitor induced cytotoxicity.

### Hematopoietic Colony Forming Cell Assay

The hematopoietic colony forming cell assay was conducted based on a protocol outlined by StemCell Technologies (Vancouver, British Columbia, Canada). To begin, CD34^+^ cells were isolated from residual normal bone marrow transplant specimens obtained after informed consent. Next, several concentrations of Aurora A Inhibitor I, Hesperadin, ZM447439 and AT9283 (0.01, 0.1, 1, 10 µM) and DMSO (Sigma-Aldrich, Oakville, Ontario) (10 µM) were added to separate tubes of MethoCult (StemCell Technologies), a methylcellulose matrix containing recombinant human cytokines stem cell factor (rh SCF), granulocyte macrophage colony-stimulating factor (rh GM-CSF), interleukin-3 (rh IL-3), granulocyte colony stimulating factor (rh G-CSF) and erythropoietin (rh EPO). Following the addition of CD34^+^ cells at a final concentration of 5×10^3^ cells per dish, the tubes were vortexed and allowed to stand for 5 minutes at room temperature. Next, the MethoCult mixtures were be dispensed into 35 mm dishes (Corning Incorporated, Corning, New York) through blunt end needles (StemCell Technologies) and 5 ml syringes (BD Biosciences, Mississauga, Ontario) at a volume of 1.1 ml per dish. The medium was evenly distributed across the surface of each dish by gentle tilting and rotation. The dishes were then placed in a 150 mm tissue culture dish (BD Biosciences) containing additional 35 mm dishes with sterile water to maintain humidity. The tissue culture dish was then placed at 37°C in a humidified incubator containing 5% CO_2_ for 14 days. The number of myeloid and erythroid derived colonies in both the treated and control dishes were counted and compared.

### Preparation Of Cellular Extracts

Following desired experimental conditions, cells were harvested and centrifuged at 1200 rpm at 4°C for 5 minutes. After removal of supernatant, cells were washed in cold PBS. Following an additional centrifugation step, supernatant was removed and the pellet was re-suspended in radioimmunoprecipitation assay (RIPA) buffer (50 mM Tris-HCl (pH 8), 150 mM NaCl, 1% NP-40, 0.5% sodium deoxycholate; 0.1% sodium dodecyl sulphate (SDS)) supplemented with 1% phosphatase inhibitor (Sigma-Aldrich), 1% protease inhibitor (Sigma-Aldrich) and 1% sodium orthovanadate (Alfa Aesar, Ward Hill, Massachusetts). Samples were transferred to Eppendorf tubes, left on ice for 10 minutes, vortexed and then centrifuged at 14 000 rpm for 10 minutes.

### Immunoblotting

To begin, the protein content of the lysates was measured using the Bicinchoninic Acid (BCA) Protein Assay Kit (Pierce, Rockford, Illinois), which included a protein standard curve generated with bovine serum albumin. Appropriate volumes of samples and loading buffer (50 mM Tris-HCl (pH 6.8), 2% SDS, 10% glycerol, 1% β-mercaptoethanol, 12.5 mM EDTA, 0.02% bromophenol blue) were mixed, ensuring that equal amounts of protein were loaded (30 µg of protein per well). Samples were then resolved on SDS-PAGE using various percentages of acrylamide gels (8%–12%), depending on the proteins of interest. SDS-PAGE was run in SDS running buffer (25 mM Tris, 192 mM glycine, 0.1% SDS) and then transferred to nitrocellulose membranes in transfer buffer (48 mM Tris, 39 mM glycine, 20% (v/v) methanol) at 100 volts for 2 hours at 4°C. Immunoblots were then blocked in 5% skim milk in tris-buffered saline with 0.1% Tween-20 (TBS-T; 50 mM Tris-HCl, pH 7.5, 150 mM NaCl, 0.1% (v/v) Tween-20 (Sigma-Aldrich)) for 1 to 2 hours. Immunoblots were then incubated with selected primary antibodies diluted in TBS-T with 0.1% gelatin (Bio-Rad, Mississauga, Ontario) and 0.05% sodium azide (Sigma-Aldrich) overnight. Primary antibodies included: Aurora-A (Cell Signalling, Danvers, Massachusetts; 1∶1000), Aurora-B (Cell Signalling, 1∶1000), phospho-Aurora-A/B/C (Cell Signalling, 1∶1000), caspase 7 (Cell Signalling, 1∶1000), PARP (Cell Signalling, 1∶2000) and β-Actin (Sigma-Aldrich, 1∶10000). Following washes with TBT-T 4 times for 8 to 10 minutes, immunoblots were incubated with appropriate secondary antibodies conjugated to horseradish peroxidise (Sigma-Aldrich) diluted in TBS-T plus 5% skim milk at a ratio of 1∶5000 for 2 hours at room temperature. After an additional round of washes in TBS-T, immunoblots were exposed to combined enhanced chemiluminescence (ECL) reagents (Reagent 1: 100 mM Tris-HCl (pH 8.5), 5.4 mM hydrogen peroxide; Reagent 2: 100 mM Tris-HCl (pH 8.5), 2.5 mM luminol, 0.4 mM p-coumaric acid) for 1 minute and developed by exposure to X-ray film (Christie InnoMed, Montreal, Quebec).

### Small Interfering Rna (sirna) Transfection

Leukemia cells were plated in a 6 well plate (BD Biosciences) at a concentration of 1×10^6^ cells per well in a volume of 500 µl of Opti-MEM supplemented with FBS and antibiotics. Next, Silencer Select Negative Control Number 1 siRNA (Invitrogen), Aurora kinase A siRNA (s196, Invitrogen) and Aurora kinase B siRNA (s17611, Invitrogen) were diluted in individual tubes (BD Biosciences) to a final concentration of 30 nM in 500 µl Opti-MEM without supplements. Following, HiPerfect Transfection Reagent (Qiagen, Toronto, Ontario) was added to the diluted siRNA at a ratio of 3∶100 and the samples were left at room temperature for 10 minutes to allow for the formation of transfection complexes. The transfection complexes were then added to the cells at a volume of 500 µl per well and the plates were placed in the incubator for 6 hours. After incubation, 1.4 ml of Opti-MEM plus supplements were added to each well, for a total volume 2.4 ml per well. The cells were returned to the incubator for an additional 48 or 72 hours and then processed for immunoblotting.

## Results

### Cytotoxicity Profiling Of Aurora Kinase Inhibitors Against Pediatric And Infant Leukemia Cell Lines And Primary Samples

**Figure 1:**
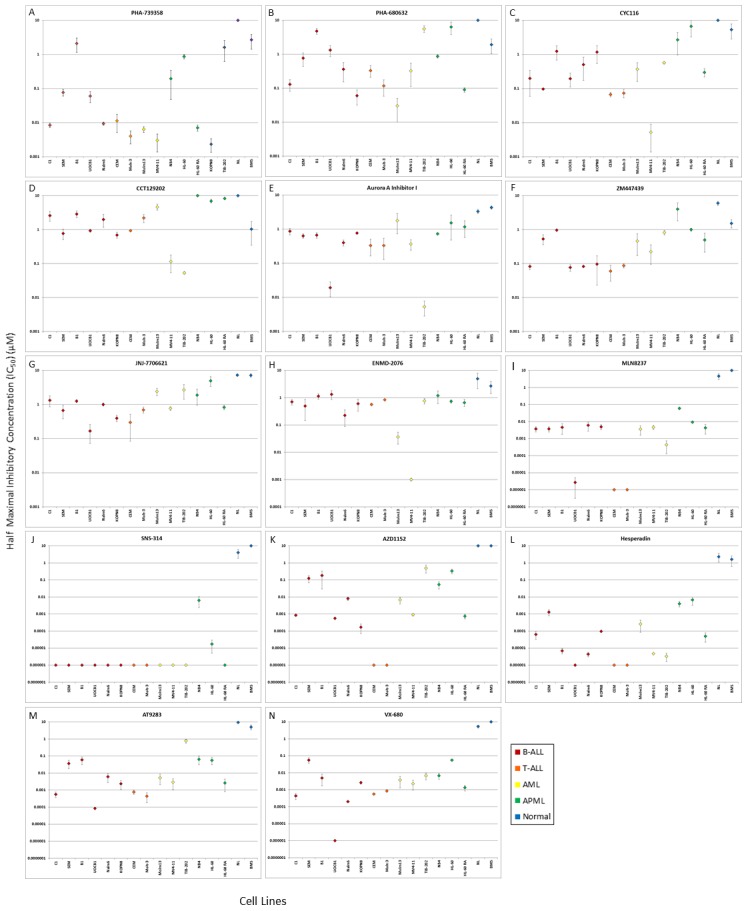
Graphical representation of the IC_50_ values of a panel of Aurora kinase inhibitors against leukemia cell lines, normal lymphocytes and bone marrow stroma presented inTable 2. The diverse sensitivity of each Aurora kinase inhibitor in this panel against B-ALL (C1, SEM, B1, UOCB1, Nalm6, KOPN8), T-ALL (CEM, Molt-3), AML (Molm13, MV4-11, TIB-202), APML (NB4, HL-60, HL-60RA) cell lines and normal lymphocytes and bone marrow stroma (BMS) is shown in graphs A-N. Each graph indicates IC_50_ values in a tested range from 1×10^−6^ to 10 µM. Points at 1×10^−6^ µM represents IC_50_ values of 1×10^−6^ µM or less and points at 10 µM represents IC_50_ values of 10 µM or more. Values are calculated averages from three separate experiments ± standard deviations.

In order to investigate the effects of Aurora kinase inhibition on pediatric and infant leukemia, a panel of 14 Aurora kinase inhibitors, listed and described in Table 1, was tested on cell lines and primary samples. The panel of cell lines included: six B-ALL cell lines (C1, SEM, B1, UOCB1, Nalm6, KOPN8), two T-ALL cell lines (CEM, Molt-3), three AML cell lines (Molm13, MV4-11, TIB-202) and three APML cell lines (NB4, HL-60, HL-60 RA). The panel of primary samples included mononuclear fractions from pediatric patients diagnosed with B-ALL (Patient #1), relapsed AML (Patient #2), AML/CML (Patient #3), infant leukemia (Patient #4) and ALL which later relapsed as AML (secondary AML) (Patient #5). Normal lymphocytes and bone marrow stroma were employed as control samples for non-leukemia cytotoxicity. The inhibitors were tested at concentrations ranging from 1×10^−6^ to 10 µM and the generated cytotoxicity curves were used to determine the IC_50_ values for each of the inhibitors against the cell lines and primary samples. The calculated values are summarized for the cell lines, normal lymphocytes and bone marrow stroma in Table 2 and for the primary samples in Table 3. Graphical representation comparing IC_50_ values for each Aurora kinase inhibitor across cell lines and normal cells are presented in Figure 1.

**Table 1:** Summary of Aurora kinase inhibitors investigated in this study.

Inhibitor	Targets (IC_50_ Value)	References	Inhibitor	Targets (IC_50_ Value)	References
PHA-739358	Aurora-A (13 nM) Aurora-B (79 nM) Aurora-C (61 nM) c-Abl, Ret, Trk-A, FGFR-1	[44],[45]	ENMD-2076	Aurora-A (14 nM) Aurora-B (350 nM) FLT3, Src, VEGFR2, FGFR-1, c-Kit	[46],[47]
PHA-680632	Aurora-A (27 nM) Aurora-B (135 nM) Aurora-C (120 nM) FGFR-1, FLT3, LCK, Plk-1, STLK2, VEGFR2, VEGFR3	[48],[49]	MLN8237	Aurora-A (2 nM)	[50]
CYC116	Aurora-A (44 nM) Aurora-B (19 nM) Aurora-C (69 nM) VEGFR2, FLT3	[10],[51]	SNS-314	Aurora-A (9 nM) Aurora-B (31 nM) Aurora-C (3 nM) Trk-A,B, FMS, FLT4, DDR2, Axl, c-Raf	[52]–[54]
CCT129202	Aurora-A (40 nM) Aurora-B (200 nM) Aurora-C (227 nM)	[55],[56]	AZD1152	Aurora-A (1369 nM) Aurora-B (0.4 nM) Aurora-C (17 nM)	[40],[52]
Aurora A Inhibitor I	Aurora-A (3 nM) Aurora-B (3400 nM)	[57]	Hesperadin	Aurora-B (250 nM)	[58]
ZM447439	Aurora-A (110 nM) Aurora-B (130 nM)	[59]	AT9283	Aurora-A (3 nM) Aurora-B (3 nM) JAK2, FLT3, c-Abl	[22],[27]
JNJ-7706621	Aurora-A (11 nM) Aurora-B (15 nM) CDK1-4, 6	[60],[61]	VX-680	Aurora-A (36 nM) Aurora-B (18 nM) Aurora-C (25 nM) FLT3	[62]–[64]

The targets and corresponding IC_50_ values for a panel of 14 Aurora kinase inhibitors are listed.

**Table 2:** Summary of half maximal inhibitory concentration (IC_50_) values for Aurora kinase inhibitors against pediatric and infant leukemia cell lines, normal lymphocytes and bone marrow stroma.

Inhibitor	B-ALL						T-ALL	
	C1	SEM	B1	UOCB1	Nalm6	KOPN8	CEM	Molt-3
**PHA-739358**	0.008±0.001	0.08±0.02	2±0.9	0.06±0.02	0.009±9×10^−4^	0.002±0.001	0.01±0.006	0.004±0.002
**PHA-680632**	0.1±0.05	0.8±0.3	5±0.9	1±0.5	0.4±0.2	0.06±0.02	0.3±0.1	0.1±0.06
**CYC116**	0.2±0.1	0.1±0.004	1±0.6	0.2±0.09	0.5±0.3	1±0.6	0.07±0.009	0.07±0.02
**CCT129202**	3±0.8	0.8±0.3	3±0.6	0.9±0.09	2±0.8	0.7±0.1	0.9±0.05	2±0.6
**Aurora A Inhibitor I**	0.9±0.2	0.6±0.09	0.7±0.1	0.02±0.009	0.4±0.08	0.8±0.05	0.3±0.2	0.3±0.2
**ZM447439**	0.08±0.02	0.5±0.2	1±0.05	0.08±0.02	0.08±0.004	0.1±0.07	0.06±0.03	0.09±0.01
**JNJ-7706621**	1±0.5	0.7±0.3	1±0.1	0.2±0.09	1±0.1	0.4±0.08	0.3±0.2	0.7±0.1
**ENMD-2076**	0.7±0.2	0.5±0.4	1±0.3	1±0.5	0.2±0.1	0.6±0.3	0.6±0.05	0.8±0.05
**MLN8237**	0.003±0.001	0.004±0.001	0.004±0.003	3×10^−6^±2×10^−6^	0.006±0.003	0.005±0.002	<1×10^−6^	<1×10^−6^
**SNS-314**	<1×10^−6^	<1×10^−6^	<1×10^−6^	<1×10^−6^	<1×10^−6^	<1×10^−6^	<1×10^−6^	<1×10^−6^
**AZD1152**	8×10^−4^±1×10^−4^	0.1±0.06	0.2±0.1	6×10^−4^±5×10^−5^	0.008±0.002	2×10^−4^±9×10^−5^	<1×10^−6^	<1×10^−6^
**Hesperadin**	6×10^−5^±3×10^−5^	0.001±5×10^−4^	7×10^−6^±2×10^−6^	<1×10^−6^	4×10^−6^±1×10^−6^	1×10^−4^±5×10^−6^	<1×10^−6^	<1×10^−6^
**AT9283**	6×10^−4^±2×10^−4^	0.04±0.02	0.06±0.03	8×10^−5^±4×10^−6^	0.006±0.003	0.002±0.001	7×10^−4^±2×10^−4^	4×10^−4^±2×10^−4^
**VX-680**	4×10^−4^±2×10^−4^	0.06±0.02	0.005±0.003	<1×10^−6^	2×10^−4^±1×10^−5^	0.003±5×10^−4^	6×10^−4^±5×10^−5^	9×10^−4^±1×10^−4^

Inhibitor	AML			APML			Normal Lymphocytes	Bone Marrow Stroma
	Molm13	MV4-11	TIB-202	NB4	HL-60	HL-60 RA		
**PHA-739358**	0.006±0.001	0.003±0.002	2±0.9	0.2±0.1	0.9±0.1	0.007±0.001	>10	3±1
**PHA-680632**	0.03±0.02	0.3±0.2	6±1	0.9±0.09	6±2	0.09±0.01	>10	2±0.9
**CYC116**	0.4±0.2	0.005±0.004	0.6±0.05	3±2	7±3	0.3±0.08	>10	5±2
**CCT129202**	5±0.9	0.1±0.06	0.05±0.005	>10	7±0.8	8±0.5	>10	1±0.7
**Aurora A Inhibitor I**	2±1	0.4±0.1	0.005±0.002	0.7±0.05	2±1	1±0.6	3±0.5	4±0.5
**ZM447439**	0.5±0.3	0.2±0.1	0.8±0.1	4±2	1±0.1	0.5±0.3	6±0.8	2±0.4
**JNJ-7706621**	2±0.6	0.8±0.09	3±1	2±0.9	5±2	0.8±0.1	7±0.5	7±0.8
**ENMD-2076**	0.04±0.02	0.001±1×10^−4^	0.8±0.2	1±0.6	0.7±0.09	0.7±0.2	5±3	3±1
**MLN8237**	0.004±0.002	0.004±0.001	4×10^−4^±3×10^−3^	0.06±0.008	0.009±9×10^−4^	0.004±0.002	5±2	>10
**SNS-314**	<1×10^−6^	<1×10^−6^	<1×10^−6^	0.006±0.004	2×10^−5^±1×10^−5^	<1×10^−6^	4±2	>10
**AZD1152**	0.007±0.003	9×10^−4^±1×10^−4^	0.5±0.2	0.05±0.02	0.3±0.09	7×10^−4^±2×10^−4^	>10	>10
**Hesperadin**	3×10^−4^±2×10^−4^	5×10^−6^±5×10^−7^	3×10^−6^±2×10^−6^	0.004±0.001	0.006±0.003	5×10^−5^±2×10^−5^	2±1	2±0.9
**AT9283**	0.005±0.003	0.003±0.002	0.8±0.2	0.06±0.03	0.05±0.02	0.003±0.002	9±1	5±2
**VX-680**	0.004±0.002	0.002±0.001	0.007±0.003	0.007±0.002	0.06±0.005	0.001±5×10^−4^	5±0.9	>10

Tumour cells from six B-ALL cell lines (C1, SEM, B1, UOCB1, Nalm6, KOPN8), two T-ALL cell lines (CEM, Molt-3), three AML cell lines (Molm13, MV4-11, TIB-202) and three APML cell lines (NB4, HL-60, HL-60 RA), as well as normal lymphocytes and bone marrow stroma, were treated with individual inhibitors at eight concentrations (1×10^−6^ to 10 µM). After four days in culture, cell viability was quantified by Alamar blue assay or automated microscopy and percent growth inhibition was determined by comparison to cells treated with DMSO control. The calculated IC_50_ values ± standard deviations are representative of three separate experiments.

**Table 3:** Summary of half maximal inhibitory concentration (IC_50_) values for Aurora kinase inhibitors against pediatric and infant leukemia patient samples.

Inhibitor	Patient #1	Patient #2	Patient #3	Patient #4	Patient #5
	B-ALL	Relapsed AML	AML/CML	Infant Leukemia	ALL/AML
**PHA-739358**	9×10^−4^	0.3	>10	>10	0.001
**PHA-680632**	0.01	2	>10	>10	8×10^−4^
**CYC116**	0.01	0.07	>10	>10	4×10^−4^
**CCT129202**	0.7	0.4	5	>10	0.07
**Aurora A Inhibitor I**	0.8	1	5	8	0.006
**ZM447439**	0.02	>10	10	7	0.009
**JNJ-7706621**	1	0.7	>10	8	5×10^−4^
**ENMD-2076**	0.1	0.008	4	10	0.03
**MLN8237**	0.006	0.5	0.7	3	1×10^−4^
**SNS-314**	<1×10^−6^	5×10^−4^	4	8	<1×10^−6^
**AZD1152**	5×10^−6^	0.6	>10	>10	2×10^−5^
**Hesperadin**	4×10^−5^	3×10^−4^	3	2	1×10^−5^
**AT9283**	0.003	0.08	1	5	3×10^−4^
**VX-680**	9×10^−5^	0.1	2	1	5×10^−4^

Tumour cells from five patients representing several types of leukemia were treated with individual inhibitors at eight concentrations (1×10^−6^ to 10 µM). After four days in culture, cell viability was quantified by Alamar blue assay or automated microscopy and percent growth inhibition was determined by comparison to cells treated with DMSO control. The calculated IC_50_ values are representative of one complete study.

Under the experimental conditions used, for several of the inhibitors, namely MLN8237, SNS-314, AZD1152, Hesperadin, AT9283 and VX-680, the calculated IC_50_ values for the normal cells were higher than the IC_50_ values for the leukemia cell lines, indicating a potential therapeutic window where the inhibitors are effective with lower toxicity to normal cells. For the remaining inhibitors, including PHA-739358, PHA-680632, CYC116, CCT129202, Aurora A Inhibitor I, ZM447439, JNJ-7706621 and ENMD-2076, calculated IC_50_ values for several of the cell lines were lower than the IC_50_ values for the normal cells, but not as pronounced. Aurora kinase inhibitors also showed measurable activity against primary leukemia samples, especially against the samples from Patient #1, Patient #2 and Patient #5. The inhibitors appear to be less effective against the samples from Patient #3 and Patient #4, with higher IC50 values.

### Effect Of Aurora Kinase Inhibitors On *In Vitro* Hematopoietic Colony Formation

**Figure 2:**
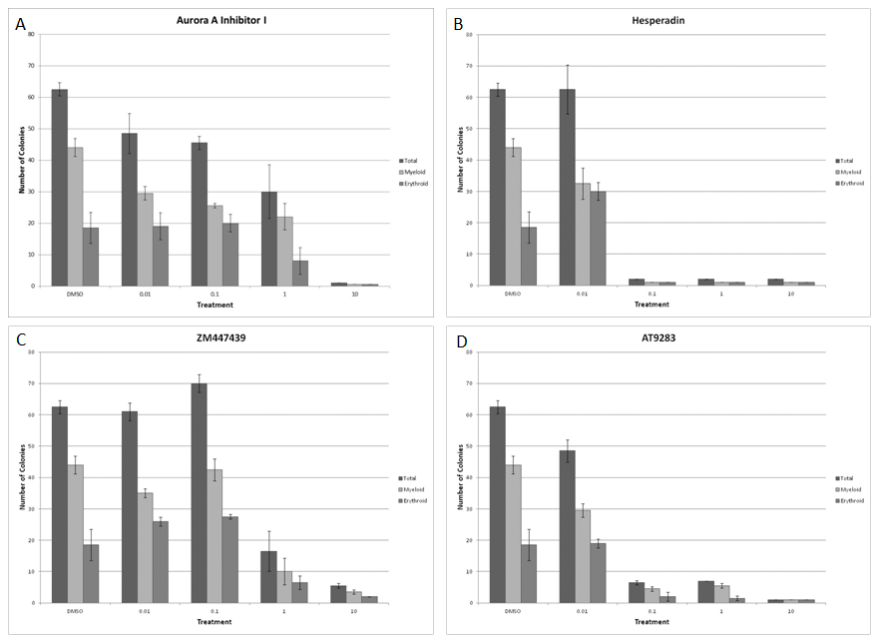
Analysis of the effect of Aurora kinase inhibitors on *in vitro* hematopoietic colony formation. Normal bone marrow derived CD34^+^ cells were incubated with increasing concentrations of Aurora A Inhibitor I (A), Hesperadin (B), ZM447439 (C), AT9283 (D) or DMSO in methylcellulose cultures containing cytokines to stimulate haematopoiesis. After 14 days in culture, myeloid and erythroid colonies were enumerated by counting under an inverted microscope based on morphology. Data presented here are representative of two separate experiments.

The colony forming assay was used to evaluate the effect of Aurora kinase inhibition on formation of multi-lineage colonies from CD34^+^ bone marrow cells *in vitro*. Increasing concentrations of four Aurora kinase inhibitors (Aurora A Inhibitor I, Hesperadin, ZM447439 and AT9283) were added to methylcellulose culture matrix containing cytokines to optimize the formation of erythroid and myeloid colonies. After 14 days in culture, colonies were counted under an inverted microscope based on morphology. Representative results presented in Figure 2 shows that colony formation in the presence of either Aurora A Inhibitor I or ZM447439 was not affected up to concentrations of 0.1 µM of the inhibitors. For Hesperadin and AT9283, colony formation remained similar to that of vehicle control plates at 0.01 µM, but completely inhibited the formation of both types of colonies at higher concentrations ranging from 0.1–10 µM.

### Aurora Kinase Inhibition By Targeted Therapeutics Promotes Changes In Aurora Kinase Activity And The Induction Of Apoptosis

**Figure 3:**
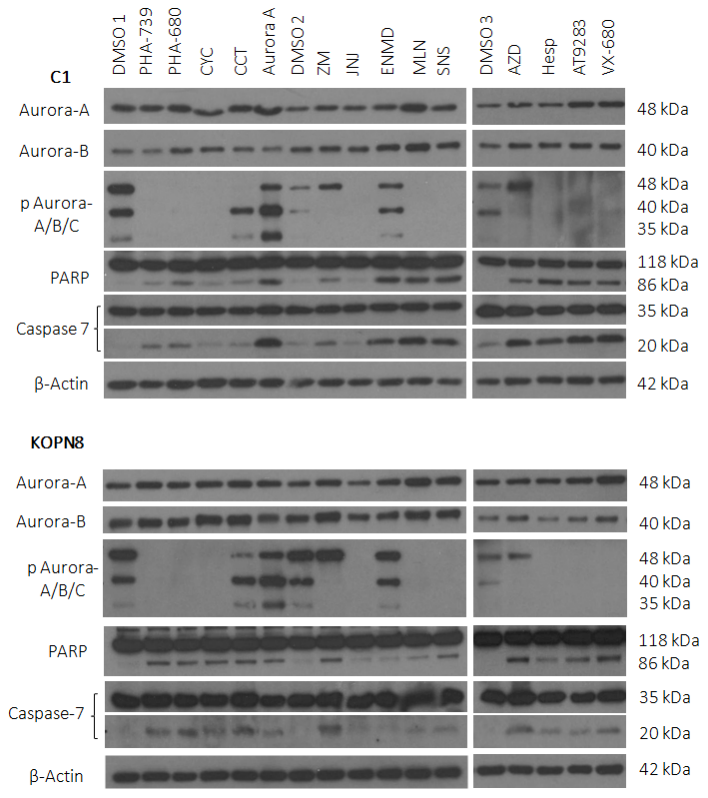
Aurora kinase inhibitors promote changes in Aurora kinase activity and induce cleavage of apoptotic markers in leukemia cell lines C1 and KOPN8. C1 and KOPN8 were treated with Aurora kinase inhibitors or vehicle control (DMSO) at 1 µM for 18 hours. The majority of the inhibitors tested induced dephosphorylation of one or more of the Aurora kinases, with the exception of Aurora-A Inhibitor I and ENMD-2076. Evidence of apoptosis is shown with increased cleavage of PARP and caspase 7 compared to vehicle control in all of the inhibitors tested, with the exception of JNJ-7706621 for both C1 and KOPN8 and ENMD-2076 for KOPN8. β-Actin was used as a loading control. Data presented are representative of three separate experiments.

Western blot analysis was performed to determine the effectiveness of Aurora kinase inhibitors at reducing Aurora kinase activity and promoting apoptosis. To begin, B-ALL cell lines C1 and KOPN8 were treated with Aurora kinase inhibitors at a concentration of 1 µM for 18 hours (Figure 3). The lysates were made in groups of four or five inhibitors plus a vehicle control for each group, designated DMSO1, DMSO2, and DMSO3. Following standard western blot protocol, it was determined that each of the inhibitors promoted dephosphorylation of one or more of the Aurora kinase isoforms compared to vehicle control, with the exception of Aurora A Inhibitor I and ENMD-2076. In addition, evidence of apoptosis was indicated by cleavage of PARP and caspase 7 by all of the inhibitors in the panel against C1 and KOPN8, with the exception of JNJ-7706621 for both cell lines and ENMD-2076 against KOPN8.

**Figure 4:**
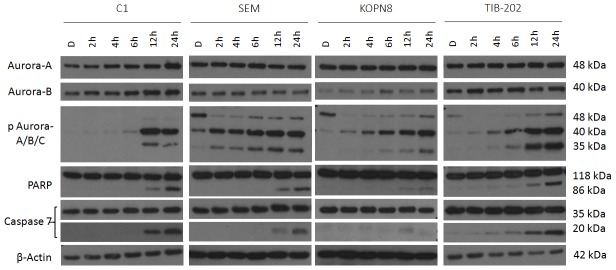
Aurora A Inhibitor I promotes changes in phosphorylation of Aurora kinases and induction of apoptosis in leukemia cell lines in a time dependent manner. C1, SEM, KOPN8 and TIB-202 were treated with 1 µM Aurora A Inhibitor I or corresponding DMSO control for 2, 4, 6, 12 or 24 hours. Western blot analysis indicates a slight increase in phosphorylation of Aurora-A at 12 and 24 hours for C1 and an initial decrease, followed by a subsequent increase in phosphorylation of Aurora-A starting at 4 hours for KOPN8 and 6 hours for both SEM and TIB-202. Both Aurora-B and Aurora-C gradually increased in phosphorylation starting at 6 hours for C1, 2 hours for SEM and KOPN8 and 4 hours for TIB-202. Induction of apoptosis is indicated by the cleavage of PARP and caspase 7 starting at 12 hours for C1, SEM and KOPN8 and 4 hours for TIB-202. β-Actin was used as a loading control. Data presented are representative of three separate experiments.

**Figure 5:**
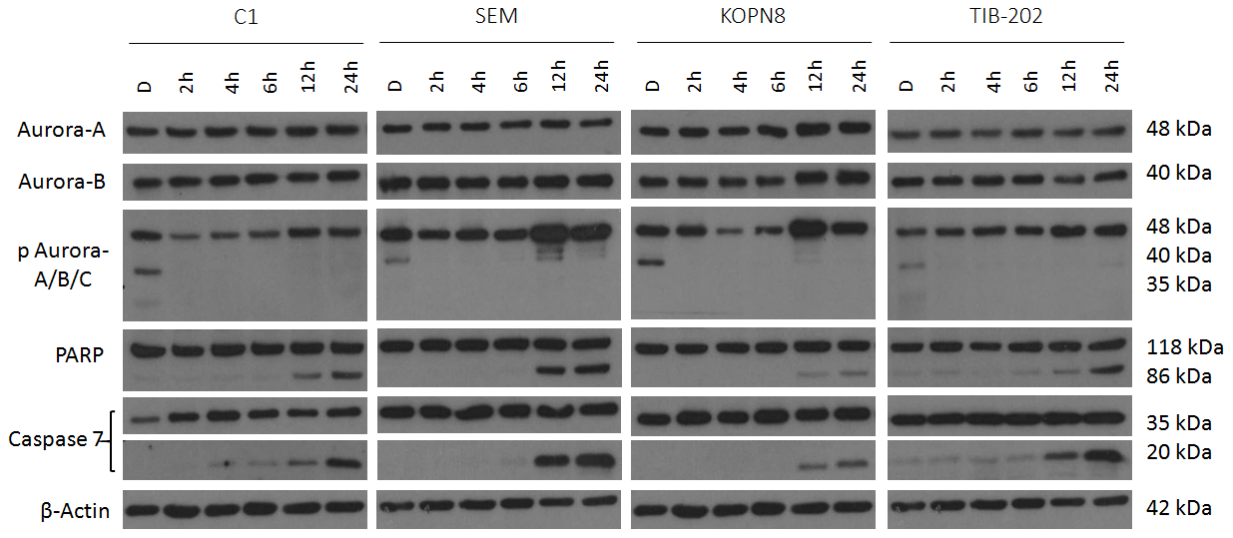
Hesperadin promotes changes in phosphorylation of Aurora kinases and induces apoptosis in leukemia cell lines in a time dependent manner. C1, SEM, KOPN8 and TIB-202 were treated with 0.1 µM Hesperadin or corresponding DMSO control for 2, 4, 6, 12 or 24 hours. Western blot analysis indicates maintained phosphorylation of Aurora-A in all cell lines from 2 to 24 hours, with a slight increase in activity starting at 12 hours. In C1 and TIB-202, dephosphorylation of Aurora-B and Aurora-C was initiated at 2 hours and maintained until 24 hours. Similarly, in SEM and KOPN8, dephosphorylation of Aurora-B was initiated at 2 hours and maintained until 24 hours. Induction of apoptosis is indicated by the cleavage of PARP starting at 12 hours and the cleavage of caspase 7 starting at 4 hours for C1 and at 12 hours for SEM, KOPN8 and TIB-202. β-Actin was used as a loading control. Data presented are representative of three separate experiments.

In order to further comprehend the differences in Aurora-A and Aurora-B function, leukemia cell lines C1, SEM, KOPN8 and TIB-202 were each treated with either Aurora A Inhibitor I (1 µM) (Figure 4) or Hesperadin (0.1 µM) (Figure 5) for 2, 4, 6, 12 and 24 hours. It was observed that Aurora A Inhibitor I induced a gradual increase in phosphorylation of Aurora-B and Aurora-C compared to vehicle control starting at 2 hours for SEM and KOPN8, 4 hours for TIB-202 and 6 hours for C1. In addition, a slight increase in phosphorylation of Aurora-A at 12 and 24 hours was observed in C1. Comparatively, an initial decrease, followed by a subsequent increase in phosphorylation of Aurora-A was apparent, starting at 6 hours for SEM and at 4 hours for KOPN8 and TIB-202. An induction of apoptosis was indicated by both the cleavage of PARP in all four cell lines starting at 12 hours and the cleavage of caspase 7 beginning at 4 hours for TIB-202 and at 12 hours for the remaining cell lines.

Following treatment of cell lines C1, SEM, KOPN8 and TIB-202 with Hesperadin, Aurora-A remained phosphorylated in all of the cell lines throughout the duration of treatment, with a slight increase in activity at 12 and 24 hours. Dephosphorylation of Aurora-B and Aurora-C was initiated at 2 hours in C1 and TIB-202 and maintained for the duration of the treatment. Similarly, dephosphorylation of Aurora-B was initiated at 2 hours in SEM and KOPN8 and maintained until 24 hours. An induction of apoptosis was indicated by both the cleavage of PARP in all four cell lines starting at 12 hours and the cleavage of caspase 7 beginning at 4 hours for C1 and at 12 hours for the remaining cell lines.

### Silencing Of Aurora Kinases By Sirna Promotes Changes In Aurora Kinase Activity And The Induction Of Apoptosis

**Figure 6:**
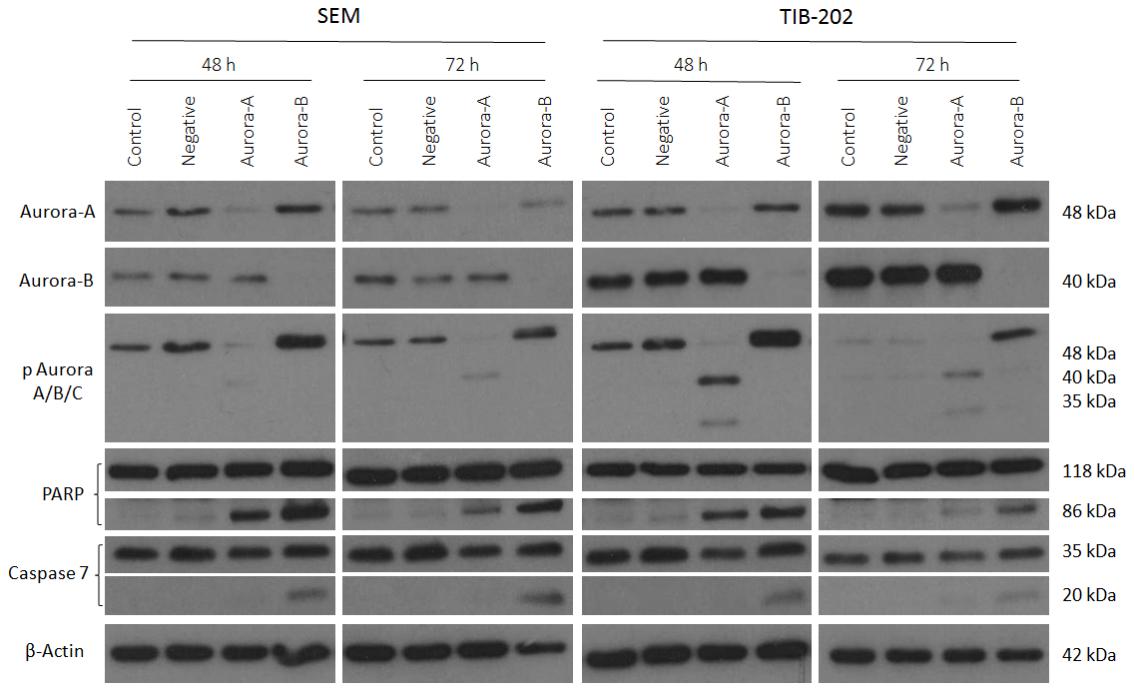
Aurora kinase siRNA promotes changes in Aurora kinase phosphorylation and cleavage of apoptotic markers in leukemia cell lines SEM and TIB-202. SEM and TIB-202 were exposed to either Aurora-A siRNA, Aurora-B siRNA or negative control siRNA for 48 and 72 hours. Silencing of Aurora-A induced increased phosphorylation of Aurora-B at 72 hours in SEM. Comparatively, silencing of Aurora-A induced increased phosphorylation of Aurora-B and Aurora-C at both time points in TIB-202. Silencing of Aurora-B induced increased phosphorylation of Aurora-A compared to controls at both 48 and 72 hours in both cell lines. Silencing of either Aurora-A or Aurora-B induced PARP cleavage at both time points. Comparatively, caspase 7 cleavage was only evident following silencing of Aurora-B. β-Actin was used as a loading control. Data presented are representative of three separate experiments.

In order to determine if the previously described changes in Aurora kinase activity and induction of apoptosis were the result of specific Aurora kinase targeting, as opposed to off-target effects or the unintended targeting of alternative proteins by these inhibitors, siRNA studies were performed. To begin, SEM and TIB-202 cells were exposed to siRNA specific to either Aurora-A or Aurora-B or a negative control siRNA for 48 and 72 hours. Cells incubated under similar conditions without siRNA were used as an additional control. As shown in Figure 6, silencing of Aurora-A in SEM induced increase phosphorylation of Aurora-B at 72 hours. In addition, silencing of Aurora-B induced increased phosphorylation of Aurora-A compared to controls at both 48 and 72 hours. With respect to apoptosis, silencing of either Aurora-A or Aurora-B induced cleavage of PARP at both time points. Comparatively, caspase 7 cleavage was only evident following silencing of Aurora-B. For TIB-202, silencing of Aurora-A induced increased phosphorylation of Aurora-B and Aurora-C at both time points. Similarly, silencing of Aurora-B induced increased phosphorylation of Aurora-A compared to controls at 48 and 72 hours. For apoptotic markers, silencing of either Aurora-A or Aurora-B induced cleavage of PARP at both time points. Comparatively, caspase 7 cleavage was only evident following silencing of Aurora-B.

### Combined Targeting Of Aurora Kinases With Sirna And Targeted Inhibitors Promotes Changes In Aurora Kinase Activity

**Figure 7:**
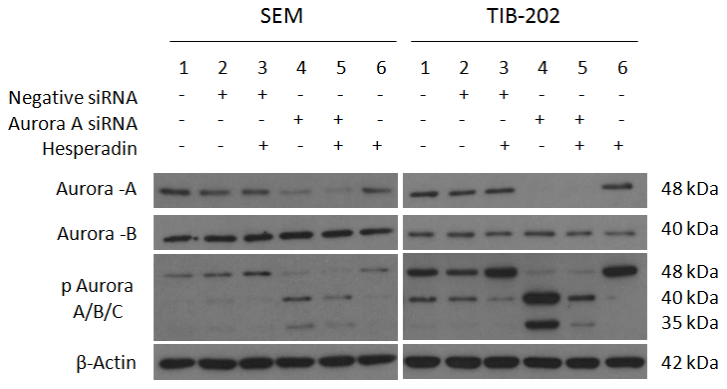
Aurora-A siRNA combined with Aurora-B inhibitor Hesperadin promotes changes in Aurora kinase phosphorylation in leukemia cell lines SEM and TIB-202. SEM and TIB-202 were exposed to either Aurora-A siRNA or negative control siRNA for 60 hours. Cells were then incubated with 0.1 µM Hesperadin or corresponding vehicle control (DMSO) for an additional 12 h. Silencing of Aurora-A promoted increased phosphorylation of Aurora-B and Aurora-C (Lane 4) and Hesperadin promoted dephosphorylation of Aurora-B (Lanes 3 and 6). With the addition of Hesperadin following silencing of Aurora-A (Lane 5), there was a decrease in phosphorylation of Aurora-B and Aurora-C compared to silencing of Aurora-A alone. Cells with no treatment and negative control siRNA were used as controls (Lanes 1 and 2). β-Actin was used as a loading control. Data presented are representative of three separate experiments.

**Figure 8:**
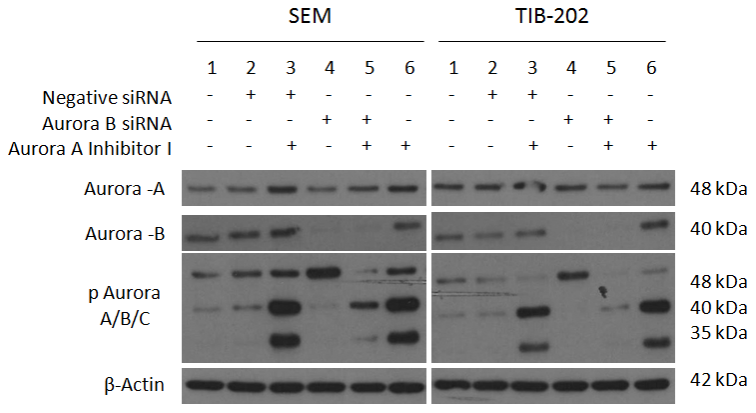
Aurora-B siRNA combined with Aurora A Inhibitor I promotes changes in Aurora kinase phosphorylation in leukemia cell lines SEM and TIB-202. SEM and TIB-202 were exposed to either Aurora-B siRNA or negative control siRNA for 60 hours. Cells were then incubated with 1 µM Aurora A Inhibitor I or corresponding vehicle control (DMSO) for an additional 12 h. Silencing of Aurora-B promoted increased phosphorylation of Aurora-A (Lane 4) in both cell lines. Aurora A Inhibitor I promoted and maintained phosphorylation of Aurora-A and induced increased phosphorylation of Aurora-B and Aurora-C in SEM (Lanes 3 and 6). Comparatively, Aurora A Inhibitor promoted dephosphorylation of Aurora-A and increased phosphorylation of Aurora-B and Aurora-C in TIB-202 (Lanes 3 and 6). With the addition of Aurora-A Inhibitor I following silencing of Aurora-B (Lane 5), there was a decrease in phosphorylation of all three isoforms compared to Aurora A Inhibitor I alone and Aurora-B siRNA alone. Cells with no treatment and negative control siRNA were used as controls (Lanes 1 and 2). β-Actin was used as a loading control. Data presented are representative of three separate experiments.

Given that silencing of Aurora-A promotes increased Aurora-B phosphorylation and silencing of Aurora-B promotes increased Aurora-A phosphorylation, experiments were conducted combining siRNA and targeted inhibitors to determine changes in Aurora kinase activity. To begin, SEM (ALL) and TIB-202 (AML) were exposed to either Aurora-A siRNA or negative control siRNA for 60 hours. Subsequently, cells were then incubated with 0.1 µM Hesperadin for an additional 12 hours. As shown in Figure 7, silencing of Aurora-A promoted increased phosphorylation of Aurora-B and Aurora-C (Lane 4) and Hesperadin promoted dephosphorylation of Aurora-B (Lanes 3 and 6). With the addition of Hesperadin following silencing of Aurora-A (Lane 5), there was a decrease in phosphorylation of Aurora-B and Aurora-C compared to silencing of Aurora-A alone. In the following experiment (Figure 8), SEM and TIB-202 were exposed to either Aurora-B siRNA or negative control siRNA for 60 hours. Cells were then incubated with 1 µM Aurora A Inhibitor I for an additional 12 h. Silencing of Aurora-B promoted increased phosphorylation of Aurora-A (Lane 4) in both cell lines. Aurora A Inhibitor I promoted and maintained phosphorylation of Aurora-A and induced increased phosphorylation of Aurora-B and Aurora-C in SEM (Lanes 3 and 6). Comparatively, Aurora A Inhibitor promoted dephosphorylation of Aurora-A and increased phosphorylation of Aurora-B and Aurora-C in TIB-202 (Lanes 3 and 6). With the addition of Aurora-A Inhibitor I following silencing of Aurora-B (Lane 5), there was a decrease in phosphorylation of all three isoforms compared to treatment with Aurora A Inhibitor I alone or Aurora-B siRNA alone.

## Discussion

Aurora kinases are overexpressed in several malignancies and are associated with aberrant mitosis and transformation [8],[16],[25]. The effect of Aurora kinase inhibition has been studied extensively in solid tumours, but information on haematological malignancies is limited, particularly in childhood leukemias. Cytotoxicity assays testing the effect of a panel of Aurora kinase inhibitors against pediatric and infant leukemia cell lines and patient samples demonstrated differences in effective inhibitory concentrations among the inhibitors (Tables 2, 3; Figure 1). There are several factors potentially responsible for these differences. These inhibitors differ structurally, inhibiting Aurora-A, Aurora-B and Aurora-C activities with varied efficiency, as indicated by their corresponding IC_50_ values (Table 1). In addition, several of these inhibitors target the activity of additional kinases, which may improve or decrease their effectiveness against malignant cells compared to normal cells. Given the diversity of pathways involved in leukemia cell growth and survival, the targeting of several kinases, particularly those overexpressed and essential to survival and/or proliferation, would be essential. Furthermore, effective responses to kinase inhibitors are influenced by additional mutations, overexpression, alterations in their downstream targets and activation of other signalling pathways [26]. We previously demonstrated the promising activity of the dual Aurora-A/B inhibitor, AT9283 against pediatric and infant leukemia, as it also targets additional proteins involved in leukemogenesis, including FLT3, JAK2 and c-Abl [27]. The application of a multi-targeted kinase inhibitor may prevent or in some instances delay the development of resistance. Despite these benefits, the lack of specificity associated with multi-targeted inhibitors has the potential to affect non-malignant cells resulting in treatment related toxicity [28]. Importantly, the distinct molecular alterations found in individual leukemia cell lines and primary samples may also contribute to the variable inhibitory effects of the Aurora kinase inhibitors. In the primary samples tested, the IC_50_ values for Patient #3 and Patient #5 appear to be affected to a lesser extent by the inhibitors. Currently, the potential mechanisms behind this observation is not known completely and thought to be multifactorial, as a similar phenomenon has been described in other targeted inhibitors and tumor models. For instance, in the melanoma model, Molhoek and colleagues have suggested that fresh tumor cells and cell lines may have differences in activation of some receptor tyrosine kinases (RTKs) and that the lower levels of activated RTKs in fresh samples may be due to deactivated RTKs in stromal cells that are often part of the specimens analysed during *in vitro* assays [29]. On the other hand, increased activation of growth supporting pathways in cell lines may result from a selected and uniform clonal population devoid of micro-environmental niche support. In addition, during preparation, fresh leukemia cells may have sustained handling stress and not yet adopted culture conditions that may have led to the expression of stress responses or artificial lowering of target availability for drugs in comparison to tumor cell lines. Consequently, any mechanism that leads to a slower rate of cell proliferation in primary specimens compared to cell lines, may potentially lead to dampening of response to Aurora kinase inhibitors. However, additional experiments are needed to clearly test these possibilities.

Western blot analysis was performed to determine the effectiveness of Aurora kinase inhibitors at reducing Aurora kinase activity and promoting apoptosis. The majority of inhibitors tested against leukemia cell lines at a concentration of 1 µM for 18 hours decreased Aurora kinase activity and promoted apoptosis, indicated by the cleavage of PARP and caspase 7 (Figures 3). The absence of these events occurring with certain inhibitors, such as Aurora-A Inhibitor and ENMD-2076, are most likely attributed to the selected dosage or duration of incubation, for which changes in protein expression could not be detected. As a means to determine changes in Aurora-A and Aurora-B specific activity over time, cell lines were treated with either Aurora-A Inhibitor I or Hesperadin for 2 to 24 hours. Following Aurora-A inhibition, there was an initial decrease in Aurora-A activity followed by a subsequent increase. In addition, a gradual increase in Aurora-B and Aurora-C activity was observed (Figure 4). Comparatively, following Aurora-B inhibition, Aurora-B activity decreased, but Aurora-A activity increased during the latter part of the time course (Figure 5). These events were also confirmed by siRNA studies, in which silencing of Aurora-A promoted increased Aurora-B activity and silencing of Aurora-B promoted increased Aurora-A activity (Figure 6). Given that silencing of Aurora-A promotes increased Aurora-B phosphorylation and silencing of Aurora-B promotes increased Aurora-A phosphorylation, experiments were conducted combining siRNA and targeted inhibitors to determine changes in Aurora kinase activity. Silencing of Aurora-A promoted increased phosphorylation of Aurora-B and Aurora-C and as a single agent, Hesperadin promoted dephosphorylation of Aurora B. With the addition of Hesperadin following silencing of Aurora-A, there was a decrease in phosphorylation of Aurora-B and Aurora-C compared to silencing of Aurora A alone (Figure 7). Similarly, with the addition of Aurora-A Inhibitor I following silencing of Aurora-B, there was a decrease in phosphorylation of all three isoforms compared to Aurora A Inhibitor I alone and Aurora-B siRNA alone (Figure 8).

Data from these studies suggest the possibility of inverse regulation of Aurora kinases, as the inhibition of one isoform leads to the up-regulation of other isoform(s). Previous studies have shown that Aurora-A and Aurora-B differ in localization, expression levels and timing of activity [15]. It is also known that Aurora-A and Aurora-B functions overlap in spindle assembly during metaphase and spindle disassembly during anaphase [30]. Specifically, inactivation of both Aurora-A and Aurora-B resulted in a significant loss of microtubules resulting from unregulated activity of microtubule depolymerases Kif18B and MCAK [31]. Although it has been established that these microtubule depolymerases are substrates of Aurora kinases, the interaction between these proteins requires further investigation as phosphorylation of different residues promote and inactivate microtubule depolymerase activity [30]. With respect to spindle disassembly, combined Aurora-A and Aurora-B inactivity resulted in the inhibition of microtubule depolymerization during anaphase, halting the movement of sister chromatids to opposite poles of the cell [32]. Although additional information is required regarding this process, it is important to note that individual targeting of Aurora-A or Aurora-B does not affect microtubule depolymerization, supporting the overlap in function of these kinases [30]. Similarly, in addition to histone H3 and CENP-A, other mitotic substrates of both Aurora-A and Aurora-B have been identified, such as CENP-E, which is important for the transport of chromosomes to the spindle equator and ensuring proper chromosomal alignment [30],[33],[34]. Since there are overlapping functions and substrates between Aurora kinase isoforms, it is possible that when one isoform is inhibited, the other isoform increases in activity to compensate for this loss of function.

These results indicate that although individual Aurora-A inhibitors (MLN8237) and individual Aurora-B inhibitors (AZD1152) have shown promising activity in *in vitro* and *in vivo* studies, the targeting of individual isoforms may result in the increased activity of other isoforms. Even though these data are suggestive of potential compensatory mechanisms among the different isoforms, additional experimental evidence is needed to show that such a process may lead to the development of resistance to Aurora kinase inhibitors. Other mechanisms of resistance have been shown to be associated with Aurora kinase inhibition, including p53 as a regulator of translation and MDR proteins, autophagic proteins involved in the lysosome dependent degradation of cytoplasmic organelles and proteins and factors involved in cellular metabolism [35]. These proteins may be considered as targets for combined therapy with Aurora kinase inhibitors to decrease the incidence of resistance, particularly in subpopulations involved in the development of relapsed leukemia. In addition, Aurora kinases in conjunction with supplementary pathways important for leukemogenesis may also be targeted, through combined therapy or multi-targeted inhibitors.

Data presented herein provide strong rationale to expand the current studies in a number of key directions. Importantly, the rational selection of Aurora kinase inhibitors in combination with chemotherapeutic agents or radiation may lead to increased cellular sensitivity to these therapeutic modalities. A number of studies have suggested the ability of Aurora kinase inhibitors to show synergy or potentiation with chemotherapies such as cytarabine [36], vorinostat [37], doxorubicin [27], vincristine [38], docetaxel [39] and daunorubicin [40]. In addition, the differential sensitivity of distinct leukemia subtypes such as high risk AML or BCR-ABL positive leukemia to Aurora kinase inhibitors should be explored further to optimize the efficacy of this family of agents [41]–[43].

In conclusion, the data presented indicate that Aurora kinase inhibitors are effective against pediatric and infant leukemia cell lines and primary samples, but may vary significantly among individual agents, possibly based on substrate availability and off-target effects. It was determined that the application of these inhibitors promotes apoptosis and when one Aurora kinase isoform is inhibited, the activity of the other isoform(s) may increase in a compensatory manner. Given the overlap in function between Aurora kinases, it is conceivable that this increase in activity may act to overcome the loss of function of the inhibited isoform, which may result in decreased cytotoxicity and potential therapy induced development of resistance. Importantly, the data presented provide rationale for further evaluation of Aurora kinases as druggable targets for therapeutics in refractory pediatric leukemia. In addition, we present evidence for the considerable variability seen among the different agents and provide impetus to explore avenues for personalized treatment options based on target modulation studies on individual patient specimens.

## Supporting Information

Table S1 **Characteristics of cell lines investigated in this study.** Pediatric and infant leukemia cell lines and leukemia cell lines with molecular abnormalities commonly seen in pediatrics are summarized. (DOCX)

Table S2 **Characteristics of primary leukemia samples investigated in this study.** Primary pediatric and infant leukemia samples representing several types of pediatric leukemias are summarized. (DOCX)
