# Supplementary material for: Aurora Kinases as Druggable Targets in Pediatric Leukemia: Heterogeneity in Target Modulation Activities and Cytotoxicity by Diverse Novel Therapeutic Agents
Source: PLoS One. 2014 Jul 21;9(7):e102741. doi: 10.1371/journal.pone.0102741 (PMC4105567; doi:10.1371/journal.pone.0102741)
Supplement: Table S1 — (DOCX) [file pone.0102741.s001.docx]

**Table S1: Characteristics of cell lines investigated in this study.** Pediatric and infant leukemia cell lines and leukemia cell lines with molecular abnormalities commonly seen in pediatrics are summarized.

| **Leukemia Type** | **Cell Line** | **Age** | **Gender** | **Additional Details** | **References** |
| --- | --- | --- | --- | --- | --- |
| **B-ALL** | B1 | 14 Y | - | Biphenotypic features of early B and myeloid lineages; t(4;11)(q21;q23) | [65] |
|  | C1 | Pediatric | Male | Pre-B ALL; Phenotype: HLA-DR^+^, CD19^+^, CD19^+^, CD20^-^ | [65] |
|  | SEM | 5 Y | Female | t(4;11)(q21;q23); MLL-AF4 with an e9-e4 fusion | [66] |
|  | UOCB1 | Pediatric | - | Pro-B-cell leukemia expressing E2A-HLF; t(17;19);  Established at St. Jude's Children's Research Hospital | [67] |
|  | Nalm6 | 19 Y | Male | Pre-B-ALL; Relapsed; t(5;12)(q31q33;p12) | [68] |
|  | KOPN8 | 3 M | Female | Pre-B-ALL; t(11;19)(q23;p13); MLL-ENL fusion gene | [68] |
| **T-ALL** | CEM | 4 Y | Female | Isolated from peripheral blood; Phenotype: CD3^+^, CD4^+^, CD5^+^, CD7^+^ | [69,70] |
|  | Molt-3 | 19 Y | Male | Relapsed; High TdT activity | [71,72] |
| **AML** | MV4-11 | 10 Y | Male | Biphenotypic B myelomonocytic leukemia; GM-CSF required to establish cell line; Internal tandem duplication of FLT3 | [73,74] |
|  | Molm13 | 20 Y | Male | AML FAB M5a; ins(11;9)(q23;p22p23); MLL-AF9 fusion gene;  Internal tandem duplication of FLT3 | [74,75] |
|  | TIB-202 | 12 M | Male | AML; t(9;11)(q23;p13); MLL-AF9 fusion gene | [76] |
| **APML** | NB4 | 20 Y | Female | Relapsed; t(15;17) (q22;q11-12) | [77] |
|  | HL-60 | 36 Y | Female | Population composed of promyelocytes, myeloblasts and granulocytes;  Amplified c-myc expression; p53 deletion | [78] |
|  | HL-60 RA | 36 Y | Female | Retinoic acid resistant; Point mutation in ligand binding domain of RAR-α | [79] |

**List of References (Table S1)**

65. Freedman MH, Grunberger T, Correa P, Axelrad AA, Dube ID, et al. (1993) Autocrine and paracrine growth control by granulocyte-monocyte colony-stimulating factor of acute lymphoblastic leukemia cells. Blood 81: 3068–3075.

66. Greil J, Gramatzki M, Burger R, Marschalek R, Peltner M, et al. (1994) The acute lymphoblastic leukaemia cell line SEM with t(4;11) chromosomal rearrangement is biphenotypic and responsive to interleukin-7. Br J Haematol 86: 275–283.

67. Inaba T, Roberts WM, Shapiro LH, Jolly KW, Raimondi SC, et al. (1992) Fusion of the leucine zipper gene HLF to the E2A gene in human acute B-lineage leukemia. Science 257: 531–534.

68. Matsuo Y, Drexler HG (1998) Establishment and characterization of human B cell precursor-leukemia cell lines. Leuk Res 22: 567–579. doi:10.1016/S0145-2126(98)00050-2.

69. Foley GE, Lazarus H, Farber S, Uzman BG, Boone BA, et al. (1965) Continuous culture of human lymphoblasts from peripheral blood of a child with acute leukemia. Cancer 18: 522–529.

70. Sandstrom PA, Buttke TM (1993) Autocrine production of extracellular catalase prevents apoptosis of the human CEM T-cell line in serum-free medium. Proc Natl Acad Sci U S A 90: 4708–4712.

71. Minowada J, Onuma T, Moore GE (1972) Rosette-forming human lymphoid cell lines. I. Establishment and evidence for origin of thymus-derived lymphocytes. J Natl Cancer Inst 49: 891–895.

72. Okamura S, Chechik BE, Lee C, Gelfand EW, Mak TW (1981) Heterogeneity of Human Thymocytes and a Malignant T-Lymphoblast Cell Line, MOLT-3. Cancer Res 41: 1664–1668.

73. Lange B, Valtieri M, Santoli D, Caracciolo D, Mavilio F, et al. (1987) Growth factor requirements of childhood acute leukemia: establishment of GM-CSF-dependent cell lines. Blood 70: 192–199.

74. Scholl S, Müller R, Clement JH, Loncarevic IF, Böhmer FD, et al. (2006) ATRA can enhance apoptosis that is induced by Flt3 tyrosine kinase inhibition in Flt3-ITD positive cells. Leuk Res 30: 633–642. doi:10.1016/j.leukres.2005.10.005.

75. Matsuo Y, MacLeod RA, Uphoff CC, Drexler HG, Nishizaki C, et al. (1997) Two acute monocytic leukemia (AML-M5a) cell lines (MOLM-13 and MOLM-14) with interclonal phenotypic heterogeneity showing MLL-AF9 fusion resulting from an occult chromosome insertion, ins(11;9)(q23;p22p23). Leukemia 11: 1469–1477.

76. Tsuchiya S, Yamabe M, Yamaguchi Y, Kobayashi Y, Konno T, et al. (1980) Establishment and characterization of a human acute monocytic leukemia cell line (THP-1). Int J Cancer J Int Cancer 26: 171–176.

77. Lanotte M, Martin-Thouvenin V, Najman S, Balerini P, Valensi F, et al. (1991) NB4, a maturation inducible cell line with t(15;17) marker isolated from a human acute promyelocytic leukemia (M3). Blood 77: 1080–1086.

78. Birnie GD (1988) The HL60 cell line: a model system for studying human myeloid cell differentiation. Br J Cancer Suppl 9: 41–45.

79. Robertson KA, Emami B, Collins SJ (1992) Retinoic acid-resistant HL-60R cells harbor a point mutation in the retinoic acid receptor ligand-binding domain that confers dominant negative activity. Blood 80: 1885–1889.
