# Supplementary material for: Aurora Kinases as Druggable Targets in Pediatric Leukemia: Heterogeneity in Target Modulation Activities and Cytotoxicity by Diverse Novel Therapeutic Agents
Source: PLoS One. 2014 Jul 21;9(7):e102741. doi: 10.1371/journal.pone.0102741 (PMC4105567; doi:10.1371/journal.pone.0102741)
Supplement: Table S2 — (DOCX) [file pone.0102741.s002.docx]

**Table S2: Characteristics of primary leukemia samples investigated in this study.** Primary pediatric and infant leukemia samples representing several types of pediatric leukemias are summarized.

| **Leukemia Type** | **Designation** | **Age** | **Gender** | **Additional Details** | **References** |
| --- | --- | --- | --- | --- | --- |
| B-ALL | Patient #1 | 13 Y | Female | 46XX; Deletion of chromosome 9 short arm; Deletion of CDKN2A;  No MLL translocations detected; WBC: 56.1/µl | - |
| Relapsed AML | Patient #2 | 15 Y | Female | t(6;9)(p23;q34); Internal tandem duplication of FLT3;  WBC: 87.8/µl (63.2% blasts) | - |
| AML/CML | Patient #3 | 11 Y | Male | 46XY, t(9;22)(q34;q11.2); WBC: 625 000/µl | - |
| Infant | Patient #4 | 6 M | Female | Pre-B ALL; 46XX, t(4;11)(q21;q23), MLL rearranged; WBC: 967 000/µl | - |
| ALL/AML | Patient #5 | 15 Y | Male | 46XY; No MLL translocations detected; WBC: 88.8/µl (76.4% blasts) | - |
